# Clinical significance of neutrophil gelatinase-associated lipocalin and sdLDL-C for coronary artery disease in patients with type 2 diabetes mellitus aged ≥ 65 years

**DOI:** 10.1186/s12933-022-01668-5

**Published:** 2022-11-17

**Authors:** Yanhong Chen, Yu Fu, Shixin Wang, Pengsheng Chen, Yunfeng Pei, Jiao Zhang, Rui Zhang, Guoping Niu, Feng Gu, Xiaoli Li

**Affiliations:** 1 Department of Clinical Laboratory, Xuzhou Central Hospital, No.199, Jiefang South Road, 221009 Xuzhou, Jiangsu, People’s Republic of China; 2 Department of Cardiology, Xuzhou Central Hospital, No.199, Jiefang South Road, 221009 Xuzhou, Jiangsu, People’s Republic of China

**Keywords:** Type 2 diabetes mellitus, Coronary artery disease, Neutrophil gelatinase-associated lipocalin, Small low-density lipoprotein, Biomarkers, Risk factors, Predicting

## Abstract

**Background and aims:**

Although type 2 diabetes mellitus (T2DM) and coronary artery disease (CAD) share many common pathological and physiological characteristics, there are few studies assessing the predictive capacity of novel biomarkers in occurrence and development of CAD in T2DM patients aged ≥ 65 years. In addition, T2DM patients aged ≥ 65 years are prone to CAD. Therefore, it is of great significance to find novel biomarkers for the development CAD in T2DM.

**Methods:**

In this retrospective cohort study, 579 T2DM patients aged ≥ 65 years were consecutively enrolled in this work, and 177 of whom had major adverse cardiovascular and cerebrovascular events (MACCE: cardiovascular or cerebrovascular death, acute coronary syndrome, coronary stent implantation, and stroke) during the follow up. Univariate and multivariate factors were employed to analyze the correlation between each variable and the occurrence of MACCE, and the Spearman’s rank correlation analysis was performed to assess the relationships between Neutrophil gelatinase-associated lipocalin (NGAL) and small dense low-density lipoprotein-cholesterol (LDL-C) (sdLDL-C). The receiver operating characteristic (ROC) curve was adopted to determine the predictive value of NGAL and sdLDL-C elevation for MACCE in T2DM patients aged ≥ 65 years.

**Results:**

After a median 48 months follow-up [19, (10 ~ 32) ], the levels of NGAL, sdLDL-C, hemoglobin A1c (HbA1c), LDL-C, and apolipoprotein B (ApoB) were significantly higher while those of high-density lipoprotein cholesterol (HDL-C) and apolipoprotein A I (ApoA–I) were lower in MACCE positive group. NGAL correlated to body mass index (BMI) (r = 0.391, P = 0.001) and triglyceride (TG) (r = 0.228, P = 0.032), and high-sensitivity CRP (hsCRP) (r = 0.251, P = 0.007), and neutrophils (r = 0.454, P = 0.001), sdlDL–C level was found to be positively correlated with LDL-C (r = 0.413, P = 0.001), TG (r = 0.432, P = 0.001), and ApoB (r = 0.232, P = 0.002); and it was negatively correlated with HDL-C (r = -0.362, P = 0.031) and ApoA–I (r = -0.402, P = 0.001). Age-adjusted Cox regression analysis showed that NGAL (HR = 1.006, 95% confidence interval (*CI*): 1.005–1.008, P < 0.001) and sdLDL–C (HR = 1.052, 95% *CI*: 1.037–1.066, P < 0.001) were independently associated with occurrence of MACCE. ROC curve analysis showed that NGAL (area under ROC (AUC) = 0.79, 95% *CI*: 0.75–0.84, *P* < 0.001) and sdlDL–C (AUC = 0.76, 95% *CI*: 0.72–0.80, *P* < 0.001) could predict the occurrence of MACCE (area under ROC. NGAL combined with sdlDL–C could predict the occurrence of MACCE well (AUC = 0.87, 95% *CI*: 0.84–0.90, *P* < 0.001).

**Conclusion:**

The higher NGAL and sdLDL-C in T2DM patients aged ≥ 65 years were significantly and independently associated with the risk of MACCE, and showed higher clinical values than other lipid biomarkers or other chronic inflammation, so they were expected to be the most effective predictors of MACCE assessment.

**Supplementary Information:**

The online version contains supplementary material available at 10.1186/s12933-022-01668-5.

## Introduction

The extensive prevalence of coronary artery disease (CAD) is a major health problem with high mortality and morbidity worldwide [1, 2]. Currently, type 2 diabetes mellitus (T2DM) affects the health and living conditions of hundreds of million people, with a global prevalence of roughly 9.3% [3]. Development of T2DM is attributed to many factors such as insulin resistance and high levels of hemoglobin A1c (HbA1c), which are also the risk factors of CAD. In addition, studies have shown that T2DM patients have a 2–3 times higher risk of CAD than healthy people, and 84% of them over 65 years old die from heart disease or stroke [4–6].

Neutrophil gelatinase-associated lipocalin (NGAL) is a 25 kD protein secreted by the activated neutrophils, adipocytes, macrophages, and other specific tissues such as prostate gland, small intestine, bronchus, pancreas, kidney, and thymus [7]. Elevated NGAL level is noticed in adipocytes when metabolic and inflammatory disorders are found [8]. NGAL is the most potential marker of acute renal injury by the Working Group of the 10th Consensus Working Meeting of the Acute Dialysis Quality Initiative (ADQI) [9]. Recent data have shown that NGAL complexes with matrix metalloproteinase-9 (MMP-9) exert structural effect, perturbing the normal physiological functions and contributing to fibrosis [10]. In addition, NGAL can be detected in atherosclerosis plaques, damaged myocardium, and arterial plaques [11, 12]. In recent years, NGAL level is proved to be related to the glucose metabolism and blood lipid composition [13], but whether it is associated with major CAD in T2DM patients aged ≥ 65 years needs further proof.

Low-density lipoprotein cholesterol (LDL–C) is a known risk factor for CAD and is the most important target for reducing the cardiovascular and cerebrovascular risk. However, the probability of predicting the risk of CAD based on a single elevated LDL-C level is about 50% [14]. LDL–C consists mainly of large and buoyant particles (lbLDL-C diameter: 20.6–22 nm), and small and dense particles (sdLDL-C diameter: 19.0–20.5 nm) [15]. sdlDL-C penetrates more through artery walls, shows a lower affinity for LDL receptors and a longer half-life in the blood, and is more easily oxidized, so it is more likely to cause arteriosclerosis [16, 17]. The sdlDL-C level is higher in the serum of CAD patients, and it is easy to susceptible to chemical modification that increases its atherogenicity [18, 19]. The National Cholesterol Education Program recognized sdLDL-C as an emerging risk factor in 2002 due to its association with CAD [20]. However, taking sdLDL-C as an independent risk factor of CAD in T2DM patients aged ≥ 65 years is still not fully matured [21, 22].

This work aimed to investigate the association between NGAL and sdlDL-C levels and major adverse cardiovascular and cerebrovascular events (MACCE: cardiovascular or cerebrovascular death, acute coronary syndrome, coronary stent implantation, and stroke) in T2DM patients aged ≥ 65 years, to evaluate the predictive value of NGAL and sdlDL-C for MACCE in these patients, and to determine whether they are more suitable than other lipid biomarkers such as LDL-C.

## Methods

### Study subjects

We performed a retrospective cohort study that was approved by the Ethics Committee of Xuzhou Central Hospital [XZXY-LJ-2016-119-070] and written informed consent was obtained from each participant. Two-sided intervals and 95% confidence were required. Individuals with T2DM were consecutively recruited from Xuzhou Central Hospital No.199, Jiefang South Road, Xuzhou 221,009, Jiangsu, People’s Republic of China from January 2017 to July 2021. Exclusion criteria included the cardio-cerebrovascular history, severe kidney and liver disease(creatinine > 2 mg/dl or ALT > 2 times upper normal limit, respectively), acute or chronic infectious disease, malignant tumor, and thyroid disease of the included subjects, and any kind of auto-immune disease, and patients with benign prostate hyperplasia or chronic respiratory disease such as asthma and COPD were also ruled out. The T2DM was diagnosed based on the international criteria 2010 issued by the World Health Organization (WHO) [23]. Dyslipidemia was defined as follows: triglyceride (TG) ≥ 150 (mg/dl), LDL-C ≥ 130 (mg/dl) or high-density lipoprotein cholesterol (HDL-C) < 40 (mg/dl) [24]. The diagnostic criteria of hypertension were defined as follows: systolic blood pressure (SBP) ≥ 140 mmhg (1 mmhg = 0.133 kPa) or diastolic blood pressure (DBP) ≥ 90 mmhg, and it should be measured 3 times to calculate the average value, or taking anti-hypertensive drugs [25]. Body mass index ( BMI ) was defined as weight in kilograms divided by the square of height in meters ( kg/m^2^ ). Oral hypoglycemic agents included insulin, other AHAs, metformin, sulfonylurea, thiazolidinedione, glinides, glitazones, α-glucosidase inhibitor and DDP-4i. All the selected individuals were reviewed in September and December 2021. The end point of this work was the first occurrence of MACCE, death, or last visit to Xuzhou Central Hospital during the follow-up period.

### Data extraction and collection

Individuals with T2DM were consecutively recruited from the Department of Clinical Laboratory, Endocrine Department and Cardiology, Xuzhou Central Hospital, No.199, Jiefang South Road, Xuzhou 221,009, Jiangsu, People’s Republic of China. The data included patients’ demographic and clinical characteristics, age, sex, ethnicity, body measurements, hypertension, medical history, and previous medications, blood content (i.e., FPG, HbA1c, Insulin, plasma TG, TC, HDL-C, LDL-C, sdlDL-C, ApoA–I, ApoB, Lp (a), hsCRP, Neutrophils, Scr, UA, NGAL), etc.

The primary outcome study was the occurrence of MACCE (major adverse cardiovascular and cerebrovascular events), including cardiovascular or cerebrovascular death, acute coronary syndrome, coronary stent implantation, and stroke. The participants were followed up every 2–4 months by phone interviews or visits at the outpatient clinic. Relevant medical information was collected during each follow-up. Follow-up data on clinical outcomes were also collected by trained independent investigators through telephonic or in-person interviews with the study participants. All patients had a total of 48 months of post-trial observation[19, (10 ~ 32) ].

### Clinical and laboratory measurements

All participants underwent a complete medical history and a comprehensive physical examination such as height, weight and blood pressure. 10-mL fasting peripheral blood was collected from each subject and centrifuged for 5 min at 3,000. After that, the serum was immediately frozen at -80 °C until testing. Serum glucose (GLU), creatinine (Scr), uric acid (UA), and lipid profiles, including total cholesterol (TC), TG, HDL-C, LDL-C, apolipoprotein A-I (ApoA-I), apolipoprotein B (ApoB), lipoprotein(a) (Lpa), and sdlDL-C were measured by standard enzymatic procedures on an automated chemical analyzer (7600-020; Hitachi, Tokyo, Japan). Glycated HbA1c concentration was measured by high-pressure liquid chromatography (Bio-Rad Inc, Hercules, CA, USA). High-sensitivity CRP (hsCRP) level was tested by particle enhanced immunoturbidimetric assay (Hitachi 917 analyzer: Boehringer Mannheim, Germany). In addition, the NGAL concentration was determined by chemiluminescence immunoassay kit (CLIA MQ60 plus; Hotgen Biotech Co., Ltd., Beijing). The Department of Clinical Laboratory of hospital implements internal and external quality control procedures.

### Statistical analysis

R Studio (embedded R3.6.3) was used for data analysis of this study. Variables with normal distribution are presented as mean ± standard deviation and variables with skewed distribution are expressed as median with inter-quartile range(IQR). Student’s t-test (for normally distributed variables) and Wilcoxon rank-sum test (for skewed variables) were used to assess the significance of differences. Categorical variables are presented as frequencies with percentages and compared via the Pearson chi-square test. The Spearman’s correlation coefficients were calculated to show the associations between NGAL or sdlDL-C with other variables; after using LASSO dimension reduction regression, potential influencing factors were screened, and collinearity among variables was removed. A multivariate COX Regression analysis was performed with study outcome and follow-up time as dependent variables, and HR was used as a risk assessment parameter, using VIF to revalidate collinearity between variables, and then using internal validation to test the robustness of regression models, using the Bootstrap sampling method, the calibration curve was drawn by repeated sampling 1000 times. A two-sided p-value of less than 0.05 meant a statistically significant difference, this test was bilateral.

## Results

### Baseline characteristics

In our study, 601 patients were included in the full analysis set. During the follow-up, there were 8 missing data, 3 missing samples, and 11 missing patients, but they showed no influence on the statistical results. Finally, 579 T2DM patients were enrolled in this work, including 369 males (63.7%) and 210 females (36.3%). All subjects were diagnosed with T2DM and the longest follow-up period was 48 months[19, (10 ~ 32) ]. During the follow-up period, there were 15 cases with cardiovascular or cerebrovascular death, 76 cases with acute coronary syndrome, 69 cases with coronary stent implantation, and 17 cases with stroke. The patients were divided into a positive group and a negative group according to whether the predefined MACCE occurred during follow-up. Compared with MACCE negative group, the proportion of anti-hypertensive therapy (P = 0.029), lipid-lowering therapy (P = 0.028), the levels of NGAL (P = 0.000), sdlDL-C (P = 0.000), HbA1c (P = 0.001), LDL-C (P = 0.000), ApoB (P = 0.002) in the positive group were significantly higher; and ApoA–I (P = 0.002) levels were lower. No significant differences were found in age (P = 0.907), gender (P = 0.653), body mass index (BMI) (P = 0.988), smoking or not (P = 0.886), hypertension (P = 0.757), dyslipidemia (P = 0.892), SBP (P = 0.055), DBP (P = 0.572), fasting plasma glucose (FPG) (P = 0.304), insulin (P = 0.513), TG (P = 0.728), TC (P = 0.959), HDL-C (P = 0.782), Lp(a) (P = 0.183), hsCRP (P = 0.970), neutrophils (P = 0.206), Scr (P = 0.931), and UA (P = 0.938) between two groups (Table 1). In this study, oral hypoglycemic agents included insulin (P = 0.131), other AHAs (P = 0.7866), metformin (P = 0.477), sulfonylurea (P = 0.619), thiazolidinedione (P = 0.852), glinides (P = 0.481), glitazones (P = 0.729), α-glucosidase inhibitor (P = 0.934), DDP-4i (P = 0.757), we have made a corresponding analysis on the use of these drugs and their impact on NGAL and sdlLDL, and outcomes of interest. Unfortunately, these drugs have no impact on NGAL and sdlLDL and their corresponding results (P > 0.05 respectively). In general, the baseline characteristics were well-matched between the two groups.

**Table 1:** Clinical, biochemical, and angiographic characteristics of study subjects in different groups

	Total	MACCE		p value
		Negative (n = 402)	Positive (n = 177)	
Age (years old)	72.10 ± 5.18	72.13 ± 5.16	72.03 ± 5.24	0.978
Male/Female	360/210	281/121	127/50	0.653
BMI (kg/m^2^)	25.11 ± 2.98	25.15 ± 2.96	25.03 ± 3.03	0.913
Smoking, n (%)	318/579 (54.9)	220/402 (54.7)	98/177 (55.3)	0.886
Hypertension, n (%)	375/579 (64.8)	262/402 (65.2)	113/177 (64.8)	0.757
Dyslipidemia (%)	510/579 (88.1)	355/402 (87.8)	157/177 (88.7)	0.892
Anti-hypertensive therapy, n (%)	340/579 (58.7)	248/402 (61.7)	92/177 (52.0)	**0.029**
Lipid-lowering therapy, n (%)	377/579 (65.1)	272/402 (67.7)	105/177 (59.3)	**0.028**
SBP (mmHg)	134 ± 17	134 ± 17	132 ± 16	0.613
DBP (mmHg)	80 ± 16	77 ± 17	81 ± 15	0.982
FPG (mg/dl)	125.60 ± 17.67	124.50 ± 16.93	127.70 ± 19.11	0.141
HbA1c (%)	6.48 ± 1.57	6.30 ± 1.60	6.89 ± 1.40	**0.001**
Insulin (mU/L)	12.15 ± 3.37	12.06 ± 3.35	12.38 ± 3.42	0.572
TG (mg/dl)	125.70 ± 43.03	125.2 ± 44.19	126.90 ± 40.26	0.911
TC (mg/dl)	218.10 ± 51.17	217.80 ± 50.38	218.80 ± 53.06	0.978
HDL-C (mg/dl)	43.10 ± 10.24	43.31 ± 10.13	42.62 ± 10.50	0.755
LDL-C (mg/dl)	117.10 ± 39.65	114.30 ± 43.51	123.40 ± 28.14	**0.037**
sdlDL-C (mg/dl)	30.07 ± 10.58	27.09 ± 9.21	36.84 ± 10.40	**0.001**
ApoA-I (mg/dl)	130.90 ± 20.22	132.70 ± 21.29	126.90 ± 16.94	**0.006**
ApoB (mg/dl)	93.06 ± 17.38	91.47 ± 17.56	96.64 ± 16.47	**0.004**
Lp (a) (mg/dl)	22.26 ± 14.33	21.83 ± 14.73	23.25 ± 13.35	0.544
hsCRP (mg/L)	6.91 ± 3.18	6.89 ± 3.12	6.96 ± 3.34	0.972
Neutrophils (× 10^9^ /L)	3.63 ± 1.03	3.65 ± 0.95	3.57 ± 1.20	0.768
Scr (µmol/L)	76.41 ± 31.04	75.82 ± 27.95	77.74 ± 37.16	0.789
UA (µmol/L)	266.50 ± 89.45	265.50 ± 90.32	268.70 ± 87.63	0.922
NGAL (ug/L)	128.4 ± 70.55	106.4 ± 49.81	178.4 ± 84.09	**0.001**

Variables with p value < 0.05 were identifed as signifcant variables and they are in bold

*N* number of patients, *BMI* Body mass index, *SBP* systolic blood pressure, *DBP* diastolic blood pressure, *FPG* Fasting plasma glucose, *HbA1c* glycated hemoglobin A1c, *TG* triglyceride, *TC* total cholesterol, *HDL-C* high-density lipoprotein-C, *LDL-C* low-density lipoprotein, *sdlDL-C* small low-density lipoprotein, *ApoA-I* Apolipoprotein A–I, *ApoB* Apolipoprotein B, *Lp(a)* lipoprotein a, *hsCRP* high-sensitivity CRP, *neutrophils* number of neutrophils, *Scr* serum creatinine, *UA* uric acid, *NGA*L neutrophil gelatinase-associated lipocalin

### Correlations of NGAL and sdlDL-C

Correlations of NGAL and sdlDL-C with other variables were searched across the whole study by Spearman’s rank correlation analysis. It was found that the NGAL was significantly positively correlated with BMI (r = 0.391, p = 0.001), TG (r = 0.228, p = 0.032), hsCRP (r = 0.251, p = 0.007), and neutrophils (r = 0.454, p = 0.001). On the other hand, sdlDL–C level was positively correlated with LDL-C (r = 0.413, p = 0.001), TG (r = 0.432, p = 0.001), and ApoB (r = 0.232, p = 0.002), and negatively correlated with HDL-C (r = -0.362, p = 0.031) and ApoA–I (r = -0.402, p = 0.001). The specific results were given in Table 2.

**Table 2:** Relationship between serum NGAL and sdlDL-C levels and other variables

Variable	NGAL		Variable	sdlDL-C	
	r	*P*		r	*P*
BMI	0.391	0.001	LDL-c	0.413	0.001
TG	0.228	0.032	TG	0.432	0.001
hsCRP	0.251	0.007	ApoB	0.232	0.002
Neutrophils	0.454	0.001	HDL-C	-0.362	0.031
			ApoA-I	-0.402	0.001

*BMI* Body mass index, *TG* triglyceride, *hsCRP* high-sensitivity CRP, *neutrophils* number of neutrophils, *LDL-C* low-density lipoprotein, *ApoB* Apolipoprotein B, *HDL-C* high-density lipoprotein-C, *ApoA-I* Apolipoprotein A–I

A total of 25 independent variables were included. As the correlation coefficient analysis among independent variables was conducted before, it was found that there was a certain correlation between different independent variables, so the dimension was reduced, the most representative high-risk predictors were screened, and LASSO regression analysis was conducted for all independent variables. With penalty coefficient λ, the coefficients of the independent variables initially included in the model are gradually compressed, and the last part of the independent variable coefficients are compressed to 0, avoiding over fitting of the model. Using 10 times cross validation of minimum criterion to identify the optimal penalty coefficient in LASSO regression model λ, When λ value continues to increase to 1 standard error, The λ is the optimal value of the model, and the final independent variables were ApoA-i, APOB, HbA1c, NGAL, sdLDL-c (Fig. S1A, B).

Next, MACCE and follow-up time were the dependent variables, while age, BMI, smoking, hypertension, dyslipidemia, SBP, DBP, FPG, HbA1c, insulin, TG, TC, HDL-C, LDL-C, sdIDL-C, ApoA-I, ApoB, Lp(a), hsCRP, neutrophils, Scr, UA, and NGAL were set as independent variables. The results showed that high levels of HbA1c (HR = 1.112, 95% *CI*: 1.006–1.228, P = 0.038), sdIDL-C (HR = 1.052, 95% *CI*: 1.037–1.066, P < 0.001), NGAL (HR = 1.006, 95% *CI*: 1.005–1.008, P < 0.001) were independent risk factors for MACCE in T2DM patients aged ≥ 65 years (Table 3).

**Table 3:** Multivariate logistic regression analysis results of factors independently associated with the occurrence of MACCE of elder T2DM patients (HR, 95% *CI*)

Variables	Univariate analysis	Multivariate analysis
Anti-hypertensive therapy	0.740(0.551–0.994)^a^	-
Lipid-lowering therapy	0.715(0.531–0.962)^a^	-
HbA1c	1.187(1.080–1.304)^b^	-
LDL-C	1.003(1.001–1.005)^b^	-
sdlDL-C	1.065(1.051–1.078)^b^	1.059 (1.046–1.072)^b^
ApoA-I	0.988(0.981–0.995)^b^	-
ApoB	1.015(1.007–1.023)^b^	-
NGAL	1.007(1.005–1.008)^b^	1.008 (1.006–1.009)^b^

^a^: P < 0.01; ^b^: P < 0.001, -: no next step analysis

*HbA1c* glycated hemoglobin A1c, *LDL-C* low-density lipoprotein, *ApoA-I* Apolipoprotein A–I, *ApoB* Apolipoprotein B, *NGAL* neutrophil gelatinase-associated lipocalin

We re-validate the robustness of the model, that was the internal validation, the calibration curves of the Cox model were drawn. The results showed that the predicted probability curve of the model fitted well with the reference probability, suggesting that the model had high accuracy(Fig S2).

### Predictive value of NGAL and sdlDL-C

**Fig. 1:**
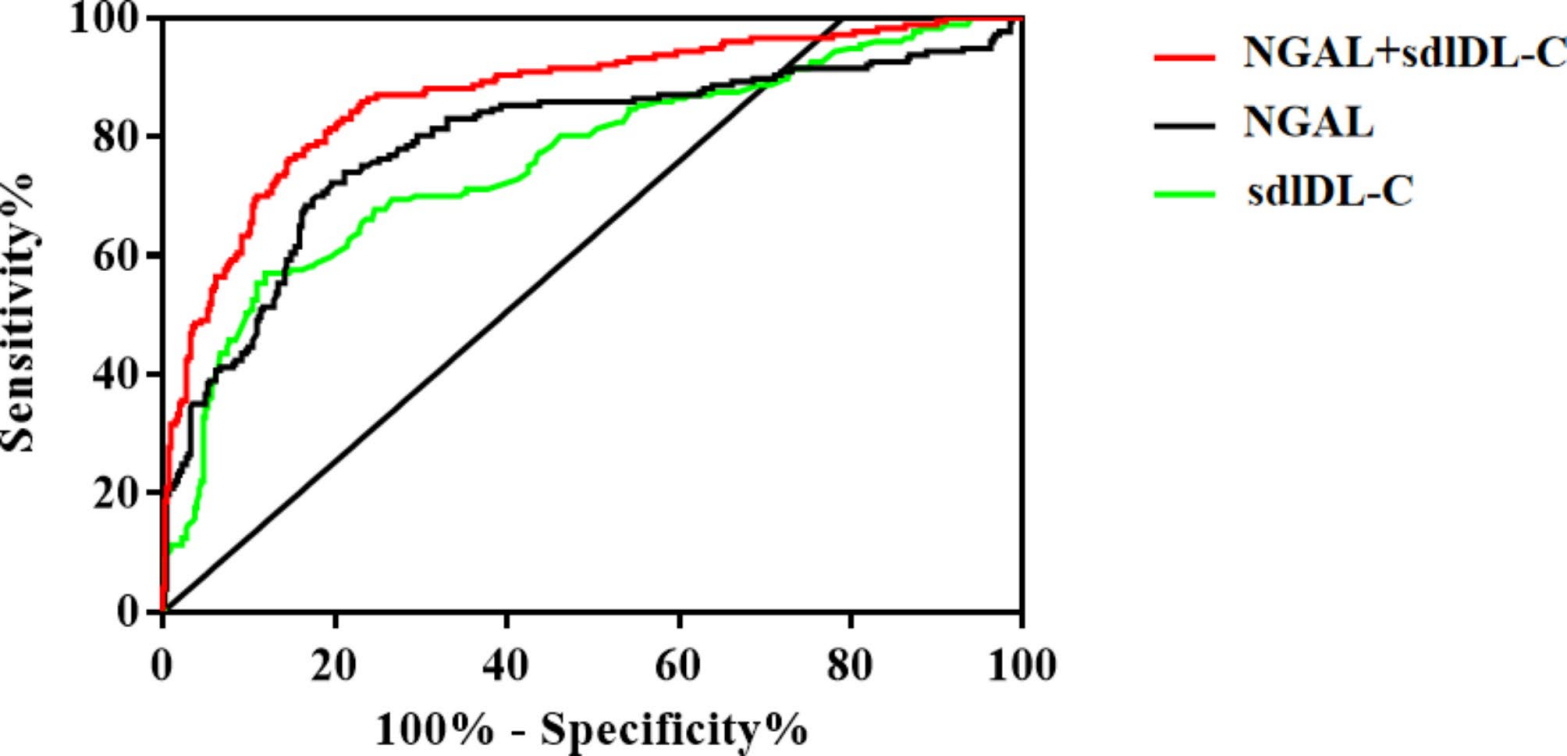
Receiver operating characteristic (ROC) analysis on the predictive capacity of the created multivariable Cox regression model for the occurrence of CAD in patients with T2DM aged ≥ 65 years. ROC analysis on the predictive capacity of the NGAL (AUC = 0.79, 95% *CI*: 0.75–0.84, *P* < 0.001) and sdlDL-C (AUC = 0.76, 95% *CI*: 0.72–0.80, *P* < 0.001) for the identification of the hazard for the primary composite outcome of major adverse cardiovascular or cerebrovascular events (MACCE: cardiovascular or cerebrovascular death, acute coronary syndrome, coronary stent implantation, and stroke), with a sensitivity of 74.01% and 69.49% and a specificity of 78.86% and 73.38%, respectively. Moreover, the combination of NGAL and sdlDL-C improved the prediction capability (AUC = 0.87, 95% CI: 0.84–0.90, *P* < 0.001)

ROC curve analysis showed that both NGAL (AUC = 0.79, 95% *CI*: 0.75–0.84, *P* < 0.001) and sdlDL-C (AUC = 0.76, 95% *CI*: 0.72–0.80, *P* < 0.001) could predict the occurrence of MACCE in T2DM patients aged ≥ 65 years. Moreover, the combination of NGAL and sdlDL-C improved the prediction capability (AUC = 0.87, 95% *CI*: 0.84–0.90, *P* < 0.001). The NGAL level of 131.9 ug/L and sdlDL-C level of 32.35 mg/dl were determined as the best cut-off points to predict the risk of MACCE of T2DM patients aged ≥ 65 years, with a sensitivity of 74.01% and 69.49% and a specificity of 78.86% and 73.38%, respectively (Fig. 1; Table 4).

**Table 4:** ROC analysis on the predictive capacity of NGAL and sdlDL-C for the occurrence of MACCE in T2DM patients aged ≥ 65 years

variable	AUC	SE	*P*	95% *CI*	Sensitivity (%)	Specificity (%)	Youden index
NGAL + sdlDL-C	0.87	0.01	< 0.001	0.84–0.90	85.88	79.07	64.95
NGAL	0.79	0.02	< 0.001	0.75–0.84	74.01	78.86	52.87
sdlDL-C	0.76	0.02	< 0.001	0.72–0.80	69.49	73.38	42.87

*ROC* receiver operating characteristic, *NGAL* neutrophil gelatinase-associated lipocalin, *sdlDL-C* small low-density lipoprotein, *MACCE* major adverse cardiovascular and cerebrovascular events, *T2DM* type 2 diabetes mellitus, *AUC* area under the curve, *SE* Std. Error, *95% CI* 95% confidence interval

## Discussion

This work demonstrated that compared with MACCE negative group in T2DM patients aged ≥ 65 years, the patients in positive group showed the higher levels of NGAL and sdlDL-C, which were associated with some other auxiliary diagnostic and therapeutic indicators such as BMI, TG, hsCRP, neutrophils, LDL-c, TG, ApoB, and ApoA-I. Moreover, it was identified in this work that advanced levels of NGAL and sdlDL-C could significantly predict the occurrence of MACCE in T2DM patients aged ≥ 65 years.

CAD and T2DM share several many common pathological and physiological characteristics. Typical cardiovascular risk factors such as dyslipidemia, hypertension, and obesity increase the risk of T2DM, and dyslipidemia is the main link between T2DM and elevated risk of CAD [26, 27]. In this work, univariate analysis showed that the higher LDL-C, HbA1c, and ApoB and lower ApoA-I and HDL-C were significantly associated with an increased CAD risk of elderly T2DM patients, suggesting that dyslipidemia and inflammatory disorders were important risk factors of CAD. Such result is consistent with that of the previous studies [8, 13].

During the development of CAD, the CRP level is elevated in addition to chronic inflammation, which is a well-established feature [28, 29], and it is positively associated with serum NGAL level even after adjustment for several confounders [30]. In this work, the number of neutrophils was found to be positively correlated with NGAL level. Similar results were obtained in the current study, suggesting that NGAL might promote the inflammatory processes leading to atherosclerosis and even MACCE. Some studies exploring the pathogenic molecular mechanisms of NGAL in atherosclerosis and CAD have shown that the NGAL/MMP-9 complexes destabilize the artery plaque, which could be detected in lipid centers and on the side facing the lumen area detected in clinical plaques. It suggests that NGAL/MMP-9 complexes are involved in vascular inflammation and reconstruction in atherosclerosis and MACCE [31].

sdlDL-C is well known to cause arteriosclerosis, which may be because that the sdLDL-C is small and deposited in the lining of the arteries and bound to glycoproteins. In addition, sdLDL-C is not easy to be cleared due to low affinity with LDL-C receptor, so it is easy to be oxidized and modified, which stimulates the macrophages to absorb lipid, forming foam cells [32]. A study has showed that the sdLDL-C level is significantly associated with the severity of CAD [33]. To the best of our knowledge, this work was the first study to evaluate the association between sdLDL-C level and the risk of MACCE in T2DM patients aged ≥ 65 years. In addition, this work proved that the combination of sdLDL-C and NGAL could effectively predict the MACCE in T2DM patients aged ≥ 65 years.

In this work, the multivariate analysis suggested that in T2DM patients aged ≥ 65 years, increased levels of NGAL and sdLDL-C were significantly associated with occurrence of MACCE. In addition, NGAL was closely correlated with BMI, TG, hsCRP, and neutrophils; and sdlDL-C level was positively correlated with LDL-c, TG, and ApoB, and negatively correlated with HDL-C and ApoA–I. Wang Y [8] confirmed that NGAL was positively correlated with gender and BMI, but not with TG, hsCRP, and neutrophils. Although NGAL is positively correlated with TG, hsCRP, and neutrophils, no established causality has been proven [34]. This work exhibited that sdLDL-C was related to MACCE lipid markers such as LDL-c, TG, and ApoB positively and to HDL-C and ApoA–I negatively. A study that comprised of 59 men with T2DM or high blood glucose has proved that the higher level of sdLDL-C was associated with body weight, age, insulin resistance, and increased intima-media thickness [35]. The sdLDL-C concentration may be a challenge risk factor in healthy people. Regardless of typical lipid tests such as TC, TG, LDL-C, and HDL-C, higher level of sdLDL-C has been detected among men and older individuals, leading to an association with greater mean carotid artery thickness [36]. Recently, some studies have found that the arterial stiffness progression in normotensive subjects could be predicted independently based on the sdLDL-C concentration. Normotensive participants with high quantiles of sdLDL-C were more likely to develop progressive arterial stiffness than those with low quantiles [37].

The results of this work suggested that elevated NGAL and sdLDL-C were significantly associated with MACCE in T2DM patients ≥ 65 years. When both NGAL and sdLDL-C were included in the multiple regression model, NGAL (HR = 1.008, 95% *CI*: 1.006–1.009, P < 0.001) and sdLDL-C (HR = 1.059, 95% *CI*: 1.046–1.072, P < 0.001) were independently associated with the risk of MACCE in T2DM patients, which was largely consistent with the above results. In addition, the ROC curve analysis showed that NGAL could predict the occurrence of MACCE in T2DM patients ≥ 65 years accurately (AUC = 0.79, 95% *CI*: 0.75–0.84, *P* < 0.001); sdlDL-C could be a strong predictor of MACCE in T2DM patients ≥ 65 years (AUC = 0.76, 95% *CI*: 0.72–0.80, *P* < 0.001); and the joint detection and prediction ability of the two was improved greatly (AUC = 0.87, 95% *CI*: 0.84–0.90, *P* < 0.001). 131.9 ug/L and 32.35 mg/dl were determined as the best cut-off points of NGAL and sdlDL-C to predict the risk of MACCE of T2DM patients aged ≥ 65 years, with sensitivity of 74.01% and 69.49% and specificity of 78.86% and 73.38%, respectively. The findings in this work could affect the featuring lipid or chronic inflammation biomarkers profiling as a useful tool in routine clinical practice in T2DM patients at high risk of CAD. Validation of novel biomarkers, offering additional prognostic value to traditional clinical and laboratory parameters, could aid clinicians in further risk-stratification in T2DM patients. This could facilitate the creation of personalized predictive algorithms, thereby expanding the concept of cardiovascular precision medicine in every-day practice.

## Limitations

This work should be explained in the context of its limitations and shortcomings. Firstly, the sample size was relatively small, and the anthropometric measures which were important indexes of CAD, obesity, and T2DM, such as waist circumference or waist-to-hip ratio, were not obtained. Secondly, to date it was not clear whether the elevated NGAL and sdLDL-C were associated with ischemic heart failure; and the efficacy of lipid-lowering, anti-diabetic, and anti-hypertensive therapy medications were not documented. Therefore, it was not possible to investigate their impacts on NGAL and sdLDL-C levels. Thirdly, it failed to search for the association between oxidative stress and higher levels NGAL and sdLDL-C during the development of MACCE of T2DM patients aged ≥ 65 years. Finally, the data used for analysis in this work derived from Chinese people with suspected or confirmed MACCE and T2DM, and hence the conclusions needed to be validated in larger clinical trials by including other European ethnicities or races to account for inherent variability of different patient populations.

## Conclusion

This work was the first one to prove that serum NGAL and sdLDL-C levels were higher in MACCE in T2DM patients aged ≥ 65 years, and the higher levels NGAL and sdLDL-C were strongly associated with some other lipid and chronic inflammation biomarkers such as LDL-C or neutrophils. After a median of 4 years of follow-up, NGAL and sdLDL-C levels were proved to be more effective predictors for risk of MACCE in T2DM patients aged ≥ 65 years.

## Electronic supplementary material: Fig. S1 and Fig. S2

Below is the link to the electronic supplementary material.

Supplementary Material 1
Fig. S1:
Lasso regression analysis. A Coefficient curves of 25 independent variables to be studied. B The best independent variable was selected by LASSO regression and 10-fold cross-validation.
Fig. S2:
Cox model calibration curve. The re-validation of the robustness of the model, i. e. the internal validation. The results show that the prediction probability curve of the model fits well with the reference probability, which indicates that the accuracy of the model is high.

## Data Availability

The datasets used and/or analyzed during the current study are available from the corresponding author on reasonable request.

## Abbreviations

T2DM: Type 2 diabetes mellitus
CAD: Coronary artery disease
MACCE: Atherosclerotic cardiovascular disease
IQR: Interquartile range
N: Number of patients
BMI: Body mass index
SBP: Systolic blood pressure
DBP: Diastolic blood pressure
FPG: Fasting plasma glucose
HbA1c: Glycated hemoglobin A1c
TG: Triglyceride
TC: Total cholesterol
HDL-C: High-density lipoprotein-C
LDL-C: Low-density lipoprotein
sdlDL-C: Small low-density lipoprotein
ApoA–I: Apolipoprotein A–I
ApoB: Apolipoprotein B
Lp(a): Lipoprotein a
hsCRP: High-sensitivity CRP
neutrophils: Number of neutrophils
Scr: Serum creatinine
UA: Uric acid
NGAL: Neutrophil gelatinase-associated lipocalin
AHAs: Antihyperglycemic agents
DPP-4i: Dipeptidyl peptidase-4 inhibitors
ROC: Receiver operating characteristic
95% *CI*: 95% confidence interval
SE: Std. Error
AUC: Area under the curve.
