# Supplementary material for: Clinical significance of neutrophil gelatinase-associated lipocalin and sdLDL-C for coronary artery disease in patients with type 2 diabetes mellitus aged ≥ 65 years
Source: Cardiovasc Diabetol. 2022 Nov 17;21:252. doi: 10.1186/s12933-022-01668-5 (PMC9682485; doi:10.1186/s12933-022-01668-5)
Supplement: Supplementary file 1 — Supplementary Material 1 Fig. S1: Lasso regression analysis. A Coefficient curves of 25 independent variables to be studied. B The best independent variable was selected by LASSO regression and 10-fold cross-validation. Fig. S2: Cox model calibration curve. The re-validation of the robustness of the model, i. e. the internal validation. The results show that the prediction probability curve of the model fits well with the reference probability, which indicates that the accuracy of the model is high. [file 12933_2022_1668_MOESM1_ESM.docx]

**Clinical significance of neutrophil gelatinase-associated lipocalin and sdLDL-C for coronary artery disease in patients with type 2 diabetes mellitus aged ≥ 65 years**


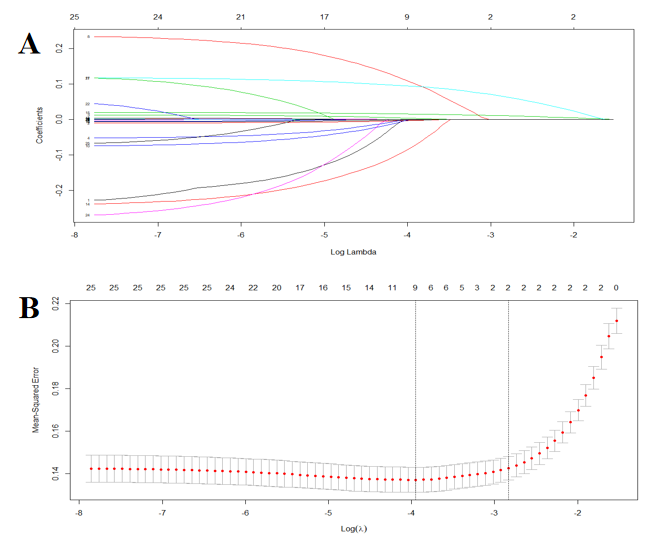


**Fig. S1** Lasso regression analysis. **A** Coefficient curves of 25 independent variables to be studied. **B** The best independent variable was selected by LASSO regression and 10-fold cross-validation.


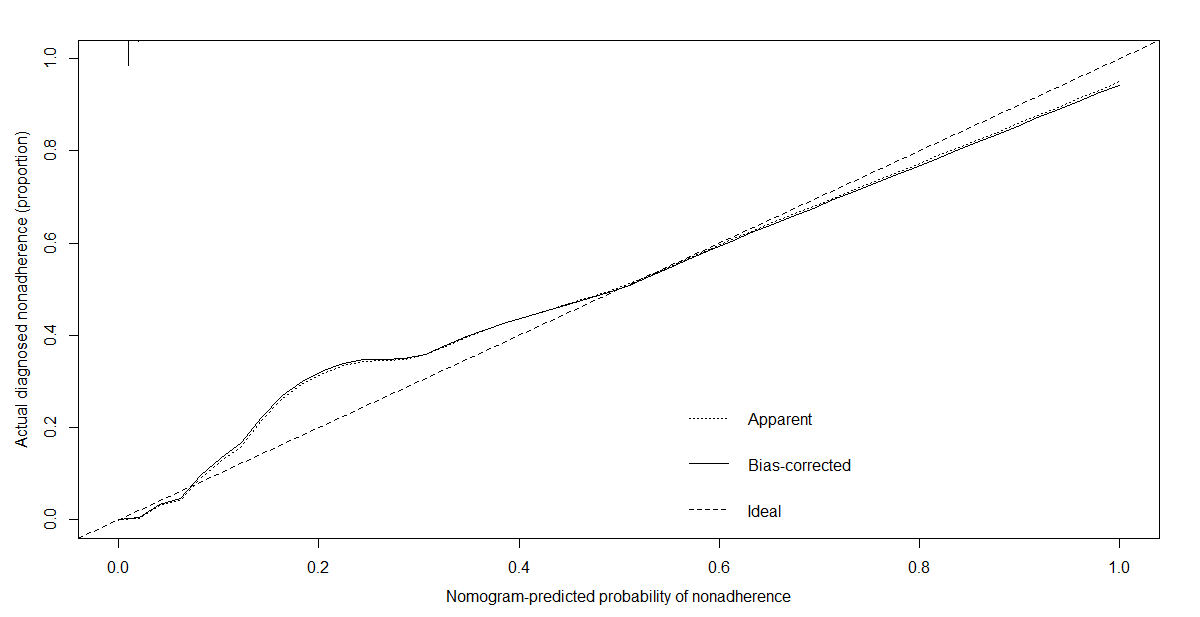


**Fig. S2** Cox model calibration curve. The re-validation of the robustness of the model, i. e. the internal validation. The results show that the prediction probability curve of the model fits well with the reference probability, which indicates that the accuracy of the model is high.
